# Resveratrol, Potential Therapeutic Interest in Joint Disorders: A Critical Narrative Review

**DOI:** 10.3390/nu9010045

**Published:** 2017-01-06

**Authors:** Christelle Nguyen, Jean-François Savouret, Magdalena Widerak, Marie-Thérèse Corvol, François Rannou

**Affiliations:** 1 Université Paris Descartes, Sorbonne Paris Cité, Paris 75006, France; jean-francois.savouret@parisdescartes.fr (J.-F.S.); marie-therese.corvol@parisdescartes.fr (M.-T.C.); francois.rannou@aphp.fr (F.R.); 2 INSERM UMR 1124, Faculté des Sciences Fondamentales et Biomédicales, Laboratoire de Pharmacologie, Toxicologie et Signalisation Cellulaire, UFR Biomédicale des Saints Pères, Paris 75006, France; 3 Service de Rééducation et de Réadaptation de l’Appareil Locomoteur et des Pathologies du Rachis, Hôpitaux Universitaires-Paris Centre, Groupe Hospitalier Cochin, Assistance Publique-Hôpitaux de Paris, Paris 75014, France

**Keywords:** trans-resveratrol, chondrocyte, synoviocytes, aryl hydrocarbon receptor, inflammation, pain, osteoarthritis, rheumatoid arthritis

## Abstract

Trans-resveratrol (t-Res) is a natural compound of a family of hydroxystilbenes found in a variety of spermatophyte plants. Because of its effects on lipids and arachidonic acid metabolisms, and its antioxidant activity, t-Res is considered as the major cardioprotective component of red wine, leading to the “French Paradox” health concept. In the past decade, research on the effects of resveratrol on human health has developed considerably in diverse fields such as cancer, neurodegenerative and cardiovascular diseases, and metabolic disorders. In the field of rheumatic disorders, in vitro evidence suggest anti-inflammatory, anti-catabolic, anti-apoptotic and anti-oxidative properties of t-Res in various articular cell types, including chondrocytes and synoviocytes, along with immunomodulation properties on T and B lymphocytes. In preclinical models of osteoarthritis and rheumatoid arthritis, resveratrol has shown joint protective effects, mainly mediated by decreased production of pro-inflammatory and pro-degradative soluble factors, and modulation of cellular and humoral responses. Herein, we comprehensively reviewed evidence supporting a potential therapeutic interest of t-Res in treating symptoms related to rheumatic disorders.

## 1. Introduction

Mediterranean societies have developed the “Mediterranean diet” under the influence of Greek medicine. This diet consists of a moderate intake of animal meat and fat in combination with high consumption of vegetables, fruits and olive oil. Natural antioxidants, fibers, B vitamins and unsaturated fatty acids are also present with red wine as the essential alcoholic beverage, consumed with meals on a daily basis [1]. The beneficial effect of this diet upon coronary heart disease prevention has been a matter of debate, recently refueled by the controversy over the “French Paradox”. This concept arose from studies showing the low rate of coronary heart disease in wine-drinking French population compared to other Western populations, despite the presence of elevated risk factors including high animal fat intake, low exercise level, and heavy smoking [2]. This controversy started on epidemiological grounds, further fueled by negative experimental data [3], and eventually led to the speculation that red wine may contain cardioprotective compounds, especially antioxidant polyphenols.

Indeed, red wine contains several polyphenols including phenolic acids, hydroxystilbenes and flavonoids. Trans-resveratrol (3,5,4′-trihydroxystilbene, t-Res) is the parent compound of a family of hydroxystilbenes existing in cis- and trans-configurations in a variety of spermatophyte plants such as grapevine, peanuts, pine or Chinese knotweed [3]. Because of its effects on lipids and arachidonic acid metabolisms, and its antioxidant activity, t-Res was considered the major cardioprotective component of red wine. In grapes, t-Res is produced as an antifungal phytoalexin in response to infection by *Bothrytis cinerea*. t-Res is present in red wine in concentrations ranging from 0.1 to 14.5 mg/L [4]. Several Asian pharmacopoeas describe Chinese knotweed (*Polygonum cuspidatum*) powder as an anti-inflammatory drug, used in various conditions such as pain, fever, dermatitis, atherosclerosis, hyperlipemia and cancers [5]. Experimental and clinical data suggest that t-Res anti-inflammatory and analgesic effects may be of clinical interest as a complementary treatment in joint diseases [4,6]. Hypotheses about resveratrol mechanisms of action suggest a modulation of endocrine disruption, apoptosis and oxidative stress. Herein, we reviewed evidence supporting a potential therapeutic interest of t-Res in treating symptoms related to rheumatic disorders. The process of article selection was unsystematic and based on authors’ expertise, self-knowledge, and reflective practice.

## 2. Literature Review

### 2.1. Molecular Targets of Resveratrol

**Figure 1:**
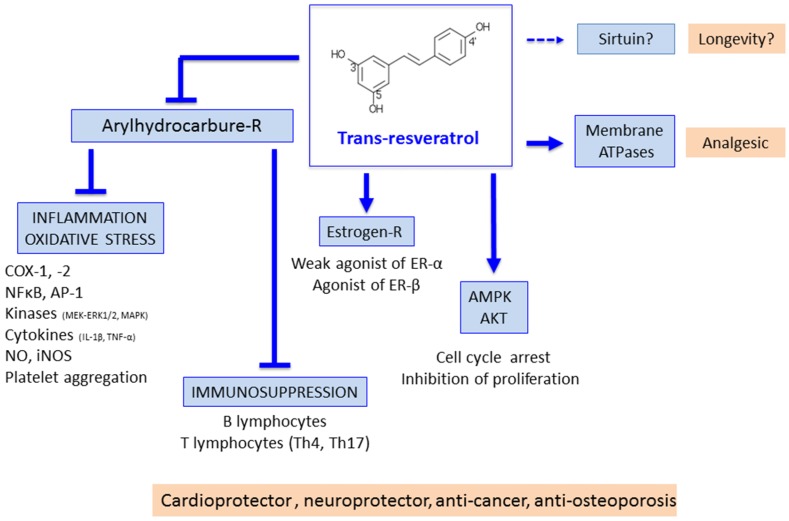
The pleiotropic effects of t-Resveratrol (t-Res). AKT: Protein kinase B; AMPK: 5' adenosine monophosphate-activated protein kinase; AP-1: Activator protein 1; COX: cyclooxygenase; ER: estrogen receptor; ERK1/2: Extracellular signal-regulated protein kinases 1 and 2; IL-1β: Interleukin-1β; MAPK: Mitogen-activated protein kinase; MEK: Mitogen-activated protein kinase kinase; NFκB: nuclear factor kappa-light-chain-enhancer of activated B cells; R: receptor; Th: T helper; TNF-α: Tumour necrosis factor-α.

Pharmacological properties of t-Res in various models have been extensively described in the literature [6,7,8,9,10,11,12]. The hypothesized beneficial properties of t-Res include detoxification through antagonization of the aryl hydrocarbon and dioxin receptor (AhR), kinases inhibition, anti-inflammatory, analgesic and anti-tumoral activities. These effects underlie the reported protective effects of t-Res in carcinogenesis, cardiovascular and neurodegenerative diseases, as well as its metabolic effects on glucose homeostasis (Figure 1).

Initial reports showed that t-Res anti-inflammatory activities, in acute and chronic phases of inflammation, were related to its ability to modulate cyclooxygenase (COX)-1 and -2 [5,13]. The first target identified at the molecular level was the AhR [14]. Aryl hydrocarbons and dioxins are found in smoke, exhaust and industrial fumes. AhR activation promotes endocrine disruption, oxidative stress, inflammation, apoptosis and immunosuppression, and is associated with an increased risk of diabetes, osteoporosis and cancers. t-Res is a strong AhR competitive antagonist (IC_50_ 6 µM) and is able to inhibit effects related to AhR activation [14]. As a stilbene, t-Res is a modulator of membrane ATPases [15], which is associated to its analgesic effects. As a polyphenol, t-Res is a weak agonist of the Estrogen Receptor (ER)-α, and an agonist of ER-β [16], a property relevant to its bone protecting effects. AhR antagonists based on the structure of t-Res, but devoid of any interaction with the ER, have been designed by our group [17]. As a polyphenol, t-Res is also a tyrosine kinases inhibitor (IC_50_ 10–25 µM), and a modulator of the MEK-ERK1/2, MAPK, AP-1 and NF-κB pathways in various tissues [18,19,20,21]. t-Res can inhibit chemical induction of pre-neoplastic lesions in mice. t-Res was proposed to act through the inhibition of COX and hydroperoxidase enzymes, by the induction of phase 2 drug-metabolizing enzymes, by its anti-oxidant activity, and by the induction of cancer cells to differentiate [22]. Cell cycle and apoptosis can also be modulated by t-Res [23,24]. More recently, the physiological role of AhR as a regulator of immune innate and adaptive responses has been evidenced [25]. The disruption of AhR leads to impaired development of immune organs and functions [26,27]. Activation of AhR by a particular ligand can induce a wide range of biological responses in Treg, Th17 and B lymphocytes, both under dioxins and t-Res influence [28,29]. This may mimic a physiological role, elicited by unknown physiological ligands.

Finally, the sirtuin (Sirt)-1 controversy has to be mentioned. A link between t-Res and Sirt-1 activation leading to cardiac function improvement in animal models of diabetic cardiomyopathy has been suggested [30]. However, whether these findings can be translated to other cardiovascular conditions remains controversial. So far, the life-expanding ability of resveratrol is limited to yeast, invertebrates and mice. Yet, mice do not live longer under resveratrol or sirtuin activator treatment, unless fed a high-fat diet. Mice diverge from humans in terms of cancer and aging. They bear an active telomerase and they never achieve “skeletal” puberty as their cartilage grows until death. Mice harbour an extremely sensitive AhR due to their lack of its endogenous negative modulator 7-keto-cholesterol [31]. In addition, the C57Bl6 mouse strain harbours an abnormal AhR with a Kd for dioxins at 6.5 pM, compared to the usual 2 to 5 nM of most mammals [32]. Could this “super” AhR produce sirtuins activators through enhanced oxidative metabolism? To date, there is no clear direct link between resveratrol and sirtuins. The addition of 5' adenosine monophosphate-activated protein kinase (AMPK) and peroxisome proliferator-activated receptor gamma coactivator (PGC)-1 in the picture might be interpreted as a drift towards the promotion of pharmaceutical drugs [33]. The doses used in vivo by Park and colleagues are 150-fold (intraperitoneally) and 3000-fold (per os) the fully active dose of 40 mg/day, when t-Res is used in the proper galenic form [34]. The doses used in Park and colleagues’ paper are in the range of 50 µM that is able to activate the ER [16] (personal data). Estradiol effects include AMPK and PGC-1 induction [35,36]. Furthermore, concerns have been raised about the recurrent trend to amalgamate resveratrol, sirtuins, calorie restriction, aging diseases and aging itself, despite righteous claims that aging is not a disease [37].

### 2.2. The Problem with Bioavailability

Animal and human studies concur on the poor bioavailability of t-Res through low uptake and extensive metabolization, notably by sulfatation and glucuronidation. This poor bioavailability is clearly a major barrier for human use. In humans and rats, t-Res rapidly undergoes conjugation resulting in one percent of the oral dose being observed as free t-Res in blood plasma [38,39,40,41]. t-Res efficiency depends on a sufficient level of active molecule in bloodstream and target tissues.

In in vitro and in vivo models, active concentrations of t-Res range from 1 to 25 µM [14,42,43]. Therefore, the development of nutritional and therapeutic application of t-Res has been greatly limited by its poor bioavailability. The active metabolites of t-Res, mainly Res-sulphate and Res-glucuronide, show higher plasma levels and are probably the only compounds that reach and accumulate in the tissues where they exert their protective effects [44]. Different strategies have been assessed to improve t-Res bioavailability [45]: (i) co-administration of t-Res metabolism inhibitors; (ii) use of naturally or chemically-synthesized t-Res analogs; and (iii) new t-Res delivery systems. t-Res bioavailability has also been improved using lipid solutions, microsomes or other vectorized forms [46]. t-Res oral dry powder forms are unable to raise t-Res plasma levels above 10 ng/mL, except for one study claiming 52 ng/mL (0.23 µM). Our group recently showed that a single dose (40 mg) of a soluble form of t-Res, in a suitable lipid vehicle, elicits micromolar concentrations in the blood of human volunteers (up to 5.7 µM; 1400 ng/mL at 30 min) [34]. t-Res was well absorbed and remained at biologically efficient blood levels for several hours, despite rapid metabolism and renal elimination. In contrast, the dry powder form was unable to elicit efficient blood levels at any time. Another benefit of the low doses used in the soluble form is the improved tolerance for t-Res. Previous dose-escalation studies in human volunteers showed that a one-gram dose of t-Res caused no significant adverse effects [38]. Higher doses were associated with infrequent minor gastrointestinal effects [47]. A study using 400 mg reported minor adverse effects such as skin rash, headache and nasopharyngitis [48]. In our study, t-Res was well tolerated and no toxicity was reported at the dose of 40 mg despite the significant increase in t-Res plasma levels [34].

### 2.3. Evidence of Joint Protective Properties

#### 2.3.1. In Vitro Findings

**Figure 2:**
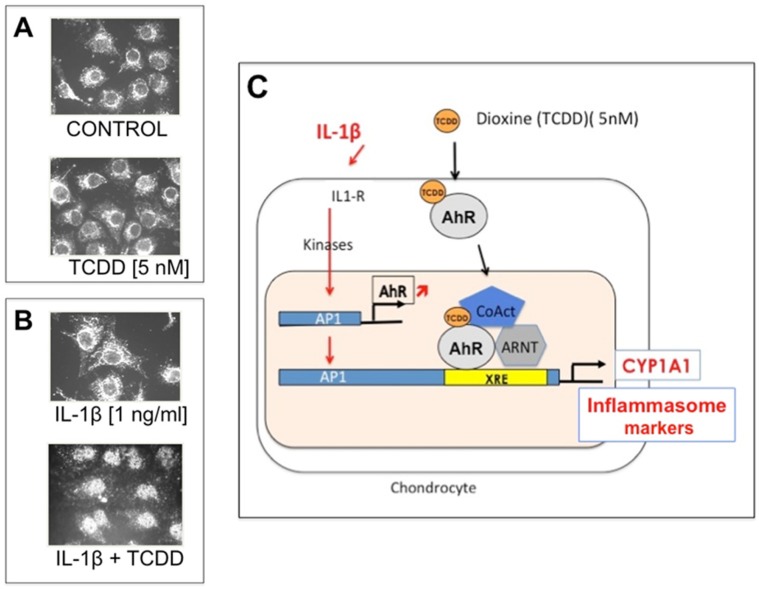
Aryl hydrocarbon and dioxin receptor (AhR)—a new target of IL-1β in chondrocytes. Rabbit chondrocytes were cultured in the absence (**A**) or in the presence (**B**) of IL-1β (5 nM) and 2,3,7,8-tetrachlorodibenzo-*p*-dioxin (TCDD). Ligand was added to the cells for 20 h. AhR expression was studied by immunocytochemistry. (**A**) In the absence of IL-1β, the AhR signal is restricted to the cytoplasmic area of chondrocytes. Addition of TCDD does not modify the AhR signal; (**B**) In contrast, in the presence of IL-1β, addition of TCDD induces AhR translocation to the nucleus; (**C**) IL-1β-increased expression of AhR over control values was shown to occur through AP1 activation and followed by robust expression of CYP1A1 (Widerak M, Corvol MT and Savouret JF, personal data).

Even though most in vitro studies reported the use of t-Res, one can assume that the cellular effects observed are rather those of active metabolites of t-Res than those of t-Res itself. Articular chondrocytes cultured in basal conditions are resistant to stimulation with active metabolites of t-Res at micromolar doses (0.1 to 10 µM) regardless of the molecular pathway considered. Several hypotheses can be brought up. Chondrocytes contain high levels of ceruleoplasmin, a copper-containing protein with a strong laccase activity [49]. Laccases are able to disrupt and inactivate active metabolites of t-Res propylene chain. Primary cultured rabbit chondrocytes also harbour particular AhR content and responsiveness to its ligands (Figure 2, Widerak M, Corvol MT and Savouret JF, personal data). In basal cultured conditions, AhR expression is weak and restricted to the cytoplasmic area of the chondrocytes (Figure 2A). In these conditions, AhR is not functional and chondrocytes do not respond to dioxin (2,3,7,8-tetrachlorodibenzo-*p*-dioxin, TCDD), the main ligand of AhR. Addition of interleukin (IL)-1β does not modify this compartmental localization. In contrast, AhR translocates to the nucleus in the presence of IL-1β plus TCDD (Figure 2B). In these conditions, increased AhR expression is observed under IL-1β stimulation upon AP1 activation, and is associated to increased expression of CYP1A1 and of inflammasome markers (Figure 2C).

**Figure 3:**
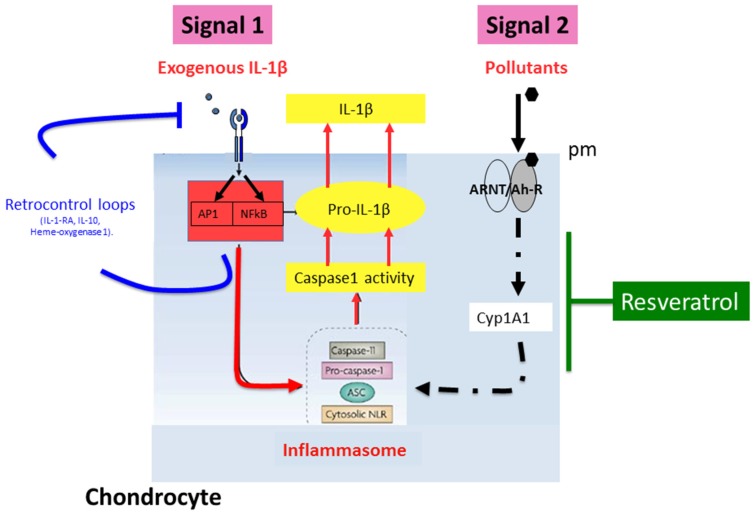
The anti-AhR effect of active metabolites of t-Res in chondrocytes could be more effective in the presence of pro-inflammatory cytokines.

The apparent inability of chondrocytes to respond to active metabolites of t-Res in basal conditions in vitro could result from the low AhR basal level, which may be under its threshold of activation. Under inflammatory conditions, such as stimulation by IL-1β, AhR content could reach such a threshold, allowing active metabolites of t-Res to inhibit AhR pathway (Figure 3). Consequently, active metabolites of t-Res could be more effective on chondrocytes in an inflammatory microenvironment. 

In addition, there is growing evidence of an effect of active metabolites of t-Res on pathological chondrocytes and synoviocytes. Inhibitory effects of active metabolites of t-Res on IL-1β and kinases modulation of matrix metalloproteinases (MMP)-1, -3, and -13 expression in chondrocytes (endochondral cartilage and intervertebral disc) have been reported by several groups [50,51,52,53,54]. In some of these studies, active metabolites of t-Res doses ranged from 50 to 100 µM. Therefore, an indirect estrogenic effect via ER-α activation cannot be excluded [55,56,57,58]. Other groups have recently opened a new avenue in cartilage research by showing that synoviocytes, more than chondrocytes, might be the real target of active metabolites of t-Res anti-inflammatory activities. Active metabolites of t-Res inhibit IL-1β, MMP-3 and phosphorylated Akt expression, either basal or Tumour Necrosis Factor (TNF) α-induced, in a dose-dependent manner, between 6 and 50 µM [59]. Caspase-8 has also been reported to be a target of active metabolites of t-Res in synoviocytes at high doses (50 µM) [60]. All the authors concluded that active metabolites of t-Res might have beneficial effects in preventing and treating rheumatoid arthritis (RA). Finally, resveratrol inhibits cell adhesion between monocytes and endothelial cells [61], a mechanism that might be extended to monocyte interactions with chondrocytes. The authors attributed this effect to tyrosine kinase inhibition, the other major effect of active metabolites of t-Res with AhR activation; although the latter mechanism is also a potent pathway for ICAM-I expression [62].

#### 2.3.2. Preclinical Findings

Although in vitro data strongly support a potent joint protection effect of resveratrol through modulation of inflammation, chondrolysis and angiogenesis, resveratrol health benefits still await in-depth investigation in animal models. Many reports use questionable modes of administration (wine, dry powder form) or erroneous targets, as once pointed out about platelet aggregation [3]. Only few papers have specifically investigated effects of t-Res in osteoarthritis (OA) or RA animal models.

In most of the studies in OA animal models, t-Res was administered by intra-articular injections. In a rabbit model of OA, by unilateral anterior cruciate ligament transection, intra-articular injections of t-Res hampered the progression of cartilage destruction and associated prod-degradative soluble factor production [63,64]. In Wang’s study, intra-articular resveratrol was administered daily for two weeks at different dose regimen (50, 20, and 10 μmol/kg). In the groups treated with resveratrol, reduced cartilage lesions, apoptosis rate of chondrocytes and level of nitric oxide in the synovial fluid were observed in a dose-dependent fashion [64]. Consistently, Elmali and colleagues showed a reduction in cartilage destruction scores and loss of matrix proteoglycans in animals injected intra-articularly with 10 μmol/kg resveratrol for two weeks compared to DMSO injected animals. Scores of synovial inflammation were comparable between the two groups [63]. Most recently, in a mouse model of OA, by destabilization of the medial meniscus, weekly intra-articular injection of resveratrol in the knee was associated with decreased cartilage and subchondral bone changes, along with unchanged type 2 collagen expression, and reduction in iNOS and MMP-13 expressions, activation of Sirt-1 and inhibition of Hypoxia Inducible Factor (HIF)-2α [65]. The chondroprotective effect of oral resveratrol supplements on C57BL/6J mice fed with a high-fat diet during 12 weeks was assessed recently. In this study, osteoarthritic histological changes, cartilage degradation assessed by levels of *C*-telopeptide of type II collagen and apoptosis assessed by TUNEL staining were reduced in mice treated with low, intermediate or high doses of oral resveratrol [66]. To our knowledge, no study has reported pain outcomes or the influence of genetic inhibition of AhR in AhR−/− mice in these models. 

Intriguingly, clinical epidemiological studies have suggested that alcohol intake, including red wine consumption, can be a protective factor in RA [67,68]. This has led to experimental studies assessing the role of red wine compounds such as resveratrol in this effect. t-Res alleviated cartilage destruction and inflammation in murine arthritis induced by collagen or lipopolysaccharides [69,70,71,72]. From a mechanistic point of view, Xuzhu and colleagues demonstrated, in the mouse collagen-induced arthritis model, that either prophylactic or therapeutic administration of resveratrol attenuated clinical arthritis parameters and bone erosions, and was associated with reduced serum levels of pro-inflammatory cytokines and collagen-specific IgG, reduced number of Th17 cells and of IL-17 in draining lymph nodes. These results suggest that the protective effects of resveratrol in this model may be mediated by the modulation of key cellular and humoral responses [72]. Most recently, Chen and colleagues, in the rat adjuvant arthritis model, reported that resveratrol (10 or 50 mg/kg) significantly reduced paw swelling and arthritis scores, with suppression of synovial hyperplasia and inflammatory cell infiltrate, and reduction in the production of COX-2 and PGE2 [69]. 

Finally, treatment with oral t-Res has shown consistent osteoprotective properties in ovariectomy-induced osteoporosis in rats [73,74]. More recently, in eight-week-old obese diabetic mouse, oral t-Res administered for three weeks was associated to increased cortical area on bone histomorphometric analysis but decreased bone length [75].

### 2.4. Interest of Resveratrol in Treating Joint Disorder-Related Symptoms in Humans

Even though research on the effect of dietary polyphenols on human health has developed considerably and controversially in the past decade in such diverse fields as cancer, neurodegenerative and cardiovascular diseases, type 2 diabetes mellitus and other metabolic disorders [76], no clinical data are available to date regarding the efficacy of resveratrol in joint disorders, especially in OA or RA. Only one randomized, double-blinded, controlled trial assessed the effect of oral resveratrol on bone mineral density and bone alkaline phosphatase in obese men of two doses (1000 and 150 mg) compared to oral placebo administered for 16 weeks. In this study, the high dose of resveratrol was associated with an increase in bone alkaline phosphatase and lumbar spine trabecular volumetric bone mineral density suggesting a potential positive effect of oral resveratrol on bone formation [77]. This lack of trials in rheumatic disorders might be related to controversies associated with the relatively poor results from resveratrol treatment in other human conditions, particularly when compared to the financial investments involved [78,79]. In a recent meta-analysis of resveratrol efficacy in type 2 diabetes mellitus, Hausenblas and colleagues found that resveratrol supplementation was more effective than placebo/control for improving systolic blood pressure, hemoglobin A1c, and creatinine [80]. However, in a broader review of all clinical trials on resveratrol performed between 2010 and 2013, Cottart and colleagues failed to confirm clear beneficial effects of resveratrol [81]. The authors pointed out concerns in comparing results from different studies because of the differences in protocols used. Furthermore, as patient groups were often small, duration of the studies often short, and/or administered doses often low, this may explain, at least in part, the lack of consistent positive results. The controversy over resveratrol clinical positive effects has been recently further fueled by Semba and colleagues who conducted a nine-year prospective cohort study in a population-based sample of 783 community-dwelling men and women 65 years or older in Italy. Their results suggest that resveratrol levels achieved with a Western diet do not have a substantial influence on health status and all-cause mortality risk [82]. However, the measurement of resveratrol exposure by 24h-urinary resveratrol metabolites only at baseline remains a matter of debate as it may not reflect the real exposure to resveratrol during the nine-year follow-up [83]. In humans, it is probably impossible, only with diet, to reach a sufficient concentration of active metabolites of t-Res at the targeted tissue. However, using food supplements that contain relatively high amounts of the compound, ranging from 55 mg to more than 500 mg per capsule, may allow reaching biologically active concentrations of the compound even considering the bioavailability factor [84].

Nevertheless, from in vitro and pre-clinical findings in the field of joint diseases, we believe that resveratrol administered with proper timing and sufficient biodisponibility, might be relevant as a complementary treatment for joint disorder-related symptoms in humans, in addition to conventional treatments.

## 3. Conclusions

For several years, based on in vitro and preclinical promising results, resveratrol has aroused keen interest because of its broad actions in almost every domain from metabolic disorders to cancer. In the field of rheumatic disorders, in vitro evidence clearly support anti-inflammatory, anti-catabolic, anti-apoptotic and anti-oxidative properties of t-Res in various articular cell types including chondrocytes and synoviocytes, along with immunomodulation properties on T and B lymphocytes. Consistently, resveratrol administered either intra-articularly or orally, has shown joint protective effects in pre-clinical models of OA and RA, mainly mediated by decreased production of pro-inflammatory and pro-degradative soluble factors, as well as modulation of cellular and humoral responses. In order to take the use of resveratrol to the next step of clinical trials in human joint diseases, new formulations of t-Res improving its biodisponibility and safety have been developed [34], and should allow for accurate assessment of its efficacy in OA and RA patients as an additional therapy to conventional treatment.
